# Design and Analysis of a Hand-Held Surgical Forceps with a Force-Holding Function

**DOI:** 10.3390/s24185895

**Published:** 2024-09-11

**Authors:** Yang Bai, Yang Yu, Zhenbang Xu

**Affiliations:** 1 Changchun Institute of Optics, Fine Mechanics and Physics, Chinese Academy of Sciences, Changchun 130033, China; by13891252266@163.com (Y.B.); xuzhenbang@ciomp.ac.cn (Z.X.); 2 Chinese Academy of Sciences Key Laboratory of On-Orbit Manufacturing and Integration for Space Optics System, Changchun 130033, China; 3 University of Chinese Academy of Sciences, Beijing 100049, China

**Keywords:** surgical forceps, kinematic analysis, stress analysis, dynamic analysis, modal optimization

## Abstract

Physiological hand tremors, twitching, and the nonlinear characteristics of the relationship between surgical forceps clamping force and operating force seriously affect the clamping accuracy of surgical instruments. To address this problem, a new type of surgical forceps with a force-holding function was developed to replace traditional forceps, which was studied in terms of structural design, statics, and dynamics. The overall structure of the surgical forceps was designed based on the lever principle, the kinematic model of the clamping part of the surgical forceps was established by the geometrical method, and the correctness of the kinematic model was verified by ADAMS. To address the clamping accuracy of the surgical forceps, a stress analysis was performed, its dynamics model was established, a finite element simulation was performed, the modal of the forceps was optimized using the Box–Behnken method, and, finally, an experimental platform was built to perform the accuracy test. The results demonstrate that the designed surgical forceps exhibit high clamping accuracy and fulfill the design specifications for surgical operations.

## 1. Introduction

Medical forceps, as a key medical device in surgery, undertake the important tasks of holding, pulling, separating, and cutting human tissues [1,2,3,4]. However, in actual surgical operations, there are involuntary movements of the surgeon’s hand, including physiological tremors, jerks, and other minute movements, which inevitably affect the precision of surgical operations [5,6,7]. In addition, the relationship between the pressure at the clamping site of medical force and operating force shows complex nonlinear characteristics and lacks a force feedback and force control module, which makes it difficult for doctors to accurately perceive the actual magnitude of the clamping force and, in turn, affects the precision of the operation of the instruments. This may lead to biomechanical compatibility and safety problems between medical force and human tissues, resulting in serious medical errors [8,9].

In order to improve the safety of surgical procedures, researchers have proposed various improvements for hand-held surgical instruments [10,11,12,13,14]. E Burdet et al. [15] designed novel haptic tweezers with an integrated six-degree-of-freedom DELTA haptic interface, which has been used for virtual reality-based microvascular surgery training. Tianci Zhang et al. [16] developed a hand-held active tremor compensation instrument to improve tip positioning accuracy in microsurgery, and the experimental results showed that for the hand-held test, the average RMS (effective value) reduction ratio was 41.0%. Christopher J. Payne et al. [17] proposed a novel microsurgical forceps with amplified force feedback and used an actuator to apply a mechanically ungrounded device to the operator’s fingertip by an amplified force. Toshihiro Kawase et al. [18] investigated the processing of a force sensor-based input device for hand-held robotic tweezers (UIA) to reduce unintended inputs in the tweezers’ tip direction operation due to gripper manipulation. Canaan Ng et al. [19] provided a one-DOF force feedback device for surgical tweezers by adding a Hall effect sensor directly to the surgical tweezers and a voice coil actuator to provide one-DOF force feedback. Latt et al. [20] developed a hand-held device with force control to maintain stable probe–tissue contact and used it for porcine rectal imaging in anal endoscopic microsurgery. Yao et al. [21] developed a haptic amplification hand-held robot to aid in lesion detection in arthroscopic interventions. A magnetic actuator was used to amplify the signal to provide vibrotactile feedback to the surgeon. Feng Ju et al. [22] investigated a miniature haptic sensor with a diameter of less than 8 mm suitable for catheterized robotic tissue hardness touching, in order to provide haptic feedback to the surgeon during surgery. Jong-Seok Oh et al. [23] proposed a novel four-DOF haptic master hand; by employing a controllable current-variable fluid, the master hand generated a four-DOF repulsive force, which had the advantages of a simple mechanism and the ability to control continuous force. Compared with medical surgical robots with other operating modes, the hand-held robot has a compact structure, low manufacturing cost, and easy integration into the normal surgical workflow [24,25,26], while retaining the sense of interaction between the surgeon’s surgical operation and the operation method of traditional surgical instruments. This greatly reduces the learning cost of the surgeon and brings a higher degree of flexibility and convenience to surgical operations [27,28].

In summary, researchers and scholars have been trying to design a solution to the problem of surgical forceps accuracy. Currently, most robotic surgical forceps solve the problem through single force feedback, but during long surgical procedures, this can lead to surgeon hand fatigue due to prolonged clamping, which can cause additional safety hazards. Meanwhile, some surgical forceps have a low fundamental frequency, which can cause resonance due to physiological hand tremors, seriously affecting the precision of surgical forceps. Compared with previous studies, this paper investigates a hand-held surgical robotic forceps with force control and a force retention function based on solving the problem of surgical forceps clamping accuracy. The fundamental frequency of the surgical forceps is much higher than the frequency of physiological hand tremors, and it has a force retention function, which is a feature that is not available in other surgical forceps. In this paper, the structural design, statics, and dynamics of the surgical forceps were investigated. The overall structure of the surgical forceps was designed, the kinematic model of the clamping part of the surgical forceps was established, and the correctness of the kinematic model was verified. A stress analysis of the surgical forceps was performed, its dynamic model was established, and finite element simulation was performed to optimize the modal state of the forceps. Finally, clamping experiments of the surgical forceps were performed, and relevant conclusions were drawn.

## 2. Materials and Structures

**Figure 1:**
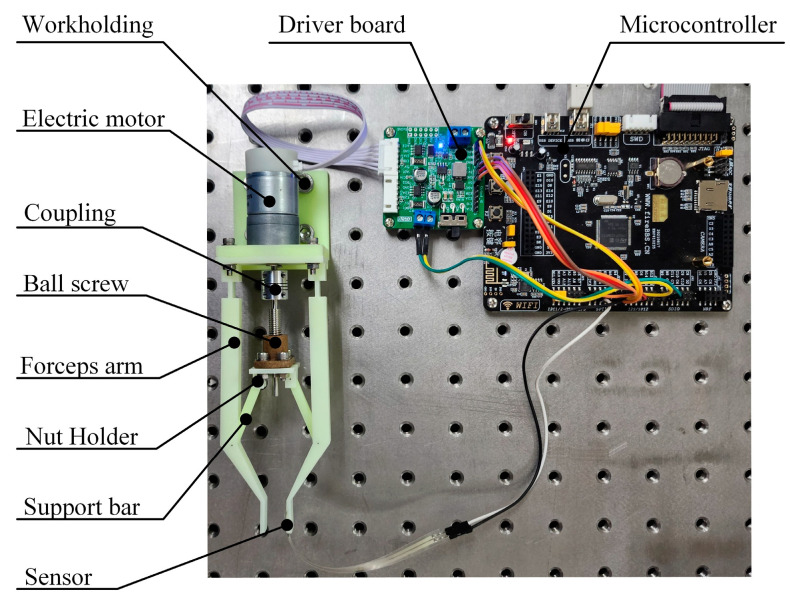
Overall structure of the surgical forceps.

The proposed forceps structure is mainly divided into an actuation unit and a control unit, as shown in Figure 1. The actuating unit mainly includes the forceps arms, a motor, a ball screw, a nut holder, and support bars. The control unit mainly includes motor drive boards and microcontrollers. The design process is based on the main principle that the overall structure size and weight are suitable for manual clamping.

The arms, nut holder, and support bar of the robot forceps are processed by Stereolithography (SLA) for lightweight, and the material is high-tenacity resin. The ball screw nut of the forceps is a miniature ball screw from MiSUMi for lightweight and miniaturization, with a lead of 1 mm. The nut is made of a special resin, and the drive unit is an MG310 motor from WHEELTEC. The MG310 motor from WHEELTEC was used for the drive, which can provide enough torque to drive the forceps to realize clamping and releasing actions. To make the forceps move according to the preset index, the MG310 motor driver board and microcontroller are used for control.

## 3. Kinematic Studies

### 3.1. Kinematic Analysis of the Forceps

**Figure 2:**
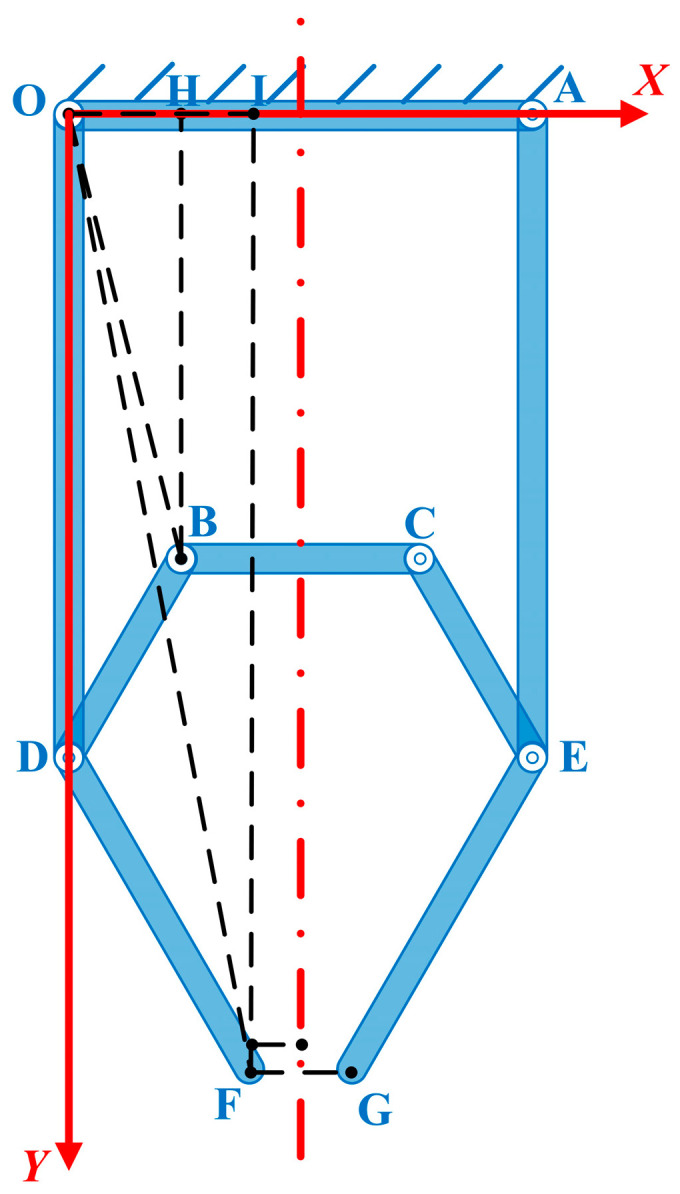
Pseudo-rigid body model of the surgical forceps.

In this paper, the kinematic model of the mechanism is developed using the pseudo-rigid body method. It is assumed that elastic deformation occurs only at the flexure, and all deformations occur in pure bending without tensile and compressive deformations. A sketch of the pseudo-rigid body model of the surgical forceps designed in this paper is shown in Figure 2.

According to the symmetry, a sketch of the surgical forceps mechanism was simplified and built (simplifying the flexible hinge into an ordinary hinge). The geometric method was used to find the displacement-positive solution for the surgical forceps, where the geometric parameters *a*, *b*, *c*, *f*, *g*, *h*, and *j* are known constants and *d*, *e*, *s*, *α*, *β*, and *γ* are variables. Let the initial values of the variables be represented by *d*_0_, *α*_0_, *β*_0_, and *γ*_0_, respectively, and the initial values are all known. *x* is the distance of the positive movement of the wire rod nut along the *Y*-axis (i.e., the distance that the rod BC moves along the *Y*-axis in the positive direction). The constitutive parameters of the surgical forceps are shown in Table 1.

In Figure 2, the relationships among the constitutive parameters of the forceps is shown in Equation (1).

(1) $$\left\{ \begin{array}{l} d=d_{0}+x\\ e=\sqrt{d^{2}+c^{2}}\\ \alpha =\arccos\frac{b^{2}+e^{2}-a^{2}}{2be}\\ \beta =\arctan\frac{d}{c}\\ \Delta \theta =\alpha +\beta -\alpha_{0}-\beta_{0}\\ \gamma =\gamma_{0}+\Delta \theta \\ \frac{s}{2}=\frac{1}{2}j-g\cos\gamma \end{array}\right.$$

Combining Equation (1), it can be seen that the distance *s* between the forceps gripping portions can be described by Equation (2).

(2) $$s=40-2g\cos\left\{\begin{array}{l} \arccos\left[\frac{{\left(d_{0}+x\right)}^{2}-a^{2}+b^{2}+c^{2}}{2b\sqrt{{\left(d_{0}+x\right)}^{2}+c^{2}}}\right]\\ +\arctan\left(\frac{d_{0}+x}{c}\right)-\frac{\pi (\alpha +\beta -\gamma )}{180} \end{array}\right\}$$

Assuming time *t* = *x*, there is a displacement equation as shown in Equation (3).

(3) $$s=40-2g\cos\left\{\begin{array}{l} \arccos\left[\frac{{\left(d_{0}+t\right)}^{2}-a^{2}+b^{2}+c^{2}}{2b\sqrt{{\left(d_{0}+t\right)}^{2}+c^{2}}}\right]\\ +\arctan\left(\frac{d_{0}+t}{c}\right)-\frac{\pi (\alpha +\beta -\gamma )}{180} \end{array}\right\}$$

It can be seen that Equation (3) is very complex, and it is obvious that the theoretical model of the velocity and acceleration of the surgical forceps will be even more complex. Therefore, MATLAB is required to determine its velocity and acceleration theoretical model. Thus, the velocity and acceleration equations of this mechanism can be simply expressed by the following inverse.

Since the velocity equation is the derivative of the displacement equation, the velocity equation is derived as in Equation (4).

(4) $$V=\frac{ds}{dt}$$

Since the acceleration equation is the second-order derivative of the displacement equation, the acceleration equation is derived as in Equation (5).

(5) $$A=\frac{d^{2}s}{dt^{2}}$$

### 3.2. Kinematic Simulation of the Forceps

**Figure 3:**
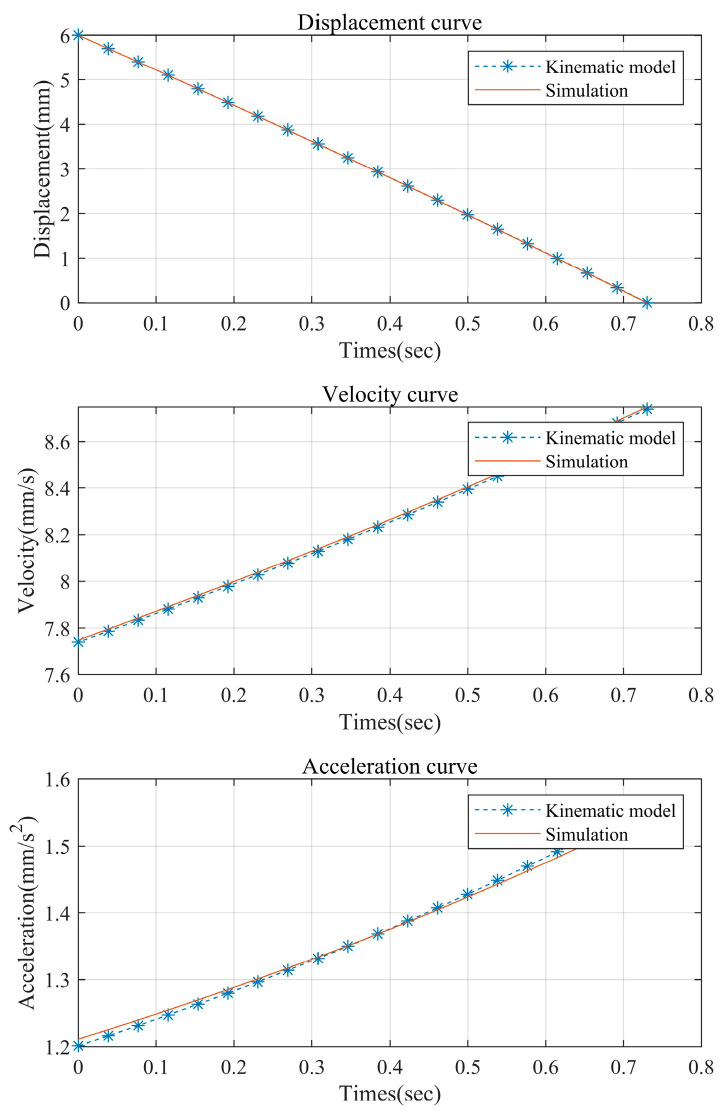
Comparison of the kinematic modeling and simulation of the clamping action.

**Figure 4:**
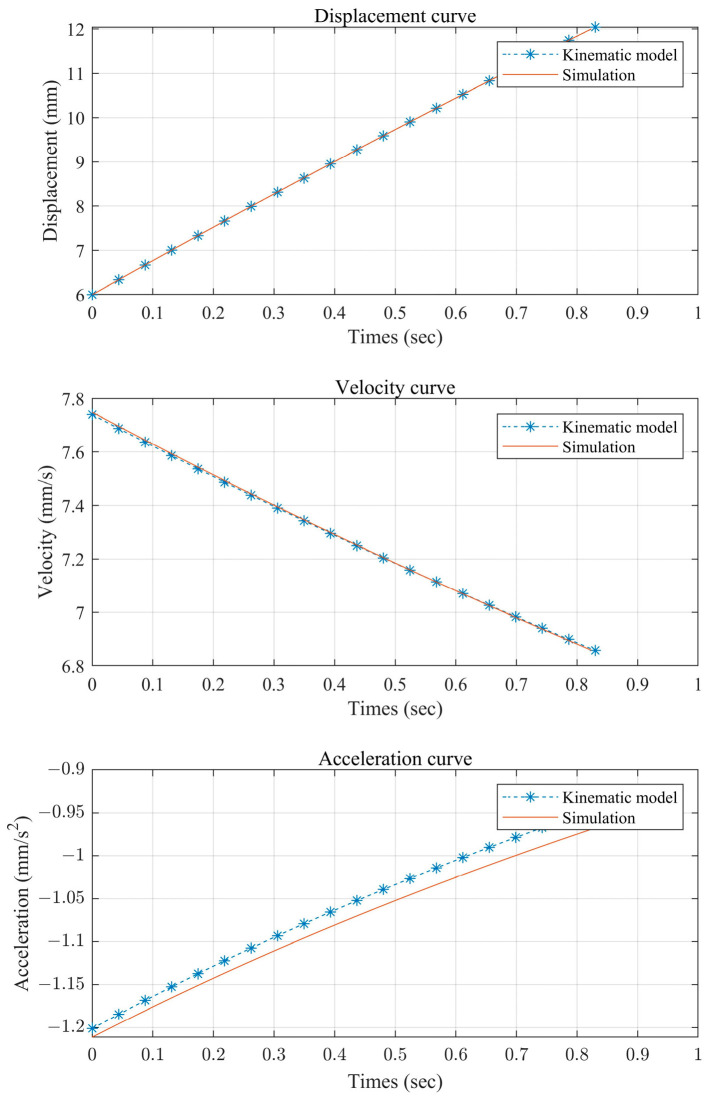
Comparison of the kinematic modeling and simulation of release action.

In order to verify the correctness of the theoretical kinematic model of the mechanism, the kinematic simulation of this mechanism is carried out in ADAMS, and the data processing is carried out using MATLAB. The comparative data plots of the theoretical kinematic model and the kinematic simulation are shown in Figure 3 and Figure 4. The reason for the error mainly lies in the pseudo-rigidification of the flexible hinge.

## 4. Dynamical Studies

In order to validate and optimize the proposed surgical forceps, stress analysis and modal optimization are carried out, while a finite element model of the forceps is developed.

### 4.1. Stress Analysis of the Forceps

**Figure 5:**
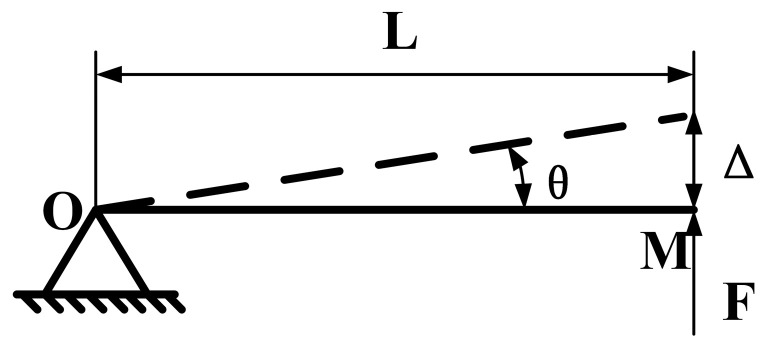
Simplified model of the arm under the forceps.

The upper and lower arms are symmetrical in the forceps structure, and it is assumed that the clamping force **F** is used to simulate the force when the forceps grasp the tissues, i.e., the model is simplified according to the knowledge of the mechanics of materials. The lower arm of the forceps is taken for this study, which is simplified as a cantilever beam that is fixed at one end and unconstrained at the other end, as shown in Figure 5.

In Figure 5, *L* is the arm length, **F** is the driving force at point *M*, and *θ* is the hinge deformation angle (forceps arm opening rotation angle). The force **F** and the maximum positive stress *σ*_max_ at the end can be obtained from the differential equation of the flexure curve in the mechanics of materials, which can be expressed by Equations (6) and (7), respectively.

(6) $$F=\frac{3EI}{L^{3}\cos\theta }$$

(7) $$\sigma_{\max}=\frac{FLy}{I\cos\theta }\leq \left[\sigma \right]$$

where *E* is the modulus of elasticity of the material (the material selected in this experiment is a high-toughness resin); *I* is the moment of inertia of the beam; Δ is one-half of the opening degree of the forceps; *y* is the distance from any point on the cross-section to the neutral axis of the cross-section (this parameter is not taken into account for the time being in this design); and [*σ*] is the maximum permissible stress of the material.

Substituting Equation (6) into Equation (7) yields Equation (8).

(8) $$\sigma_{\max}=\frac{3E\Delta y}{L^{3}\cos^{2}\theta }\leq \left[\sigma \right]$$

When the arm gripping tissue, i.e., deflection, is determined, two conclusions can be obtained from Equations (6) and (8) as follows: (1) The magnitude of the arm clamping force is equal to the external force **F** required to overcome the deformation of the arm, and the magnitude of **F** is directly proportional to the elastic modulus *E* of the arm material and inversely proportional to the length of the arm, *L*. (2) The maximum positive stress, *σ*_max_, in the arm occurs at point *O* (i.e., at the flexible hinge of the surgical forceps), which is the most prone to fracture failure.

### 4.2. Finite Element Simulation of Stress

To verify the correctness of the theoretical analysis of the designed surgical forceps, PATRAN 2010 and NASTRAN 2010 finite element analysis software was used to carry out the static analysis of the surgical forceps, which also verified the correctness of the formula. The material used for the surgical forceps is high-tenacity resin with a modulus of elasticity *E* = 2500 MPa, Poisson’s ratio *μ* = 0.3, and density *ρ* = 1.117 g/cm^3^.

**Figure 6:**
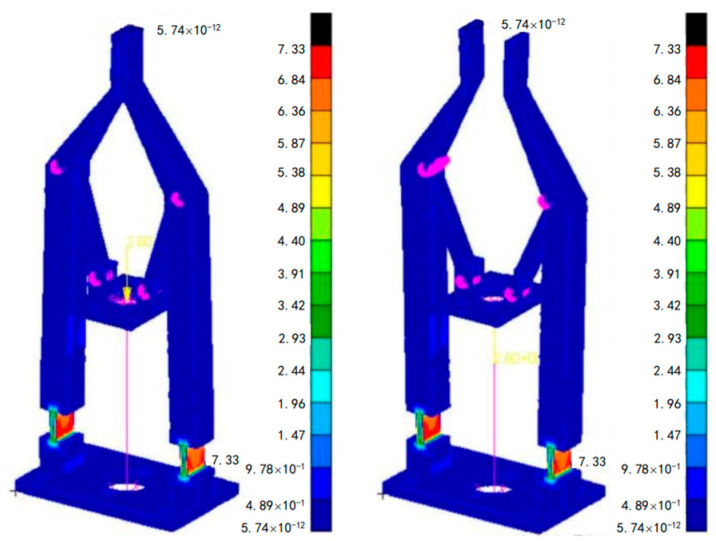
Force clouds for two limit states of the forceps.

Firstly, according to the kinematic analysis, a displacement of −0.74 mm and 0.84 mm was applied to the screw nut support plate (input end). The stress cloud of the surgical forceps is shown in Figure 6. It can be seen that the maximum stress of the surgical forceps appears at the flexible hinge; the maximum stress is much smaller than the destructive stress of the high-toughness resin material, which is consistent with the results of the stress theory analysis.

### 4.3. Stiffness Analysis of the Forceps

For multi-objective optimization of surgical forceps, it is clear from the design section that the arm of the surgical forceps is selected for optimization. The stiffness of the mechanism is theoretically analyzed as the motor, and the silk rod and other components of the surgical forceps are determined.

The driving force is mainly used to make the straight beam-type flexible hinge rotate, so its rotational stiffness needs to be calculated first. The rotational stiffness of the straight beam-type flexible hinge is shown in Equation (9).

(9) $$k=\frac{Enp^{3}}{12l}$$

where *E* is the modulus of elasticity; *n* is the depth of the hinge; *p* is the minimum thickness of the flexible hinge; and *l* is the length of the intermediate beam rod of the straight beam-type flexible hinge.

For the lower side mechanism, when the whole mechanism is driven by force and runs to perform work, the corresponding kinetic and potential energy can be obtained. Assuming that the deformation angle of the straight beam-type flexible hinge is *θ*. In Figure 2, the potential energy of the mechanism is shown in Equation (10).

(10) $$U_{1}=\frac{1}{2}k\theta^{2}$$

At the same time, Equation (11) can be obtained.

(11) $$\tan\theta =\frac{i}{h}$$

Since the amount of change in the angle of rotation of the flexible hinge is very small, it can be obtained as

(12) $$\theta =\frac{i}{h}$$

Therefore, Equation (10) can be further written as

(13) $$U_{1}=\frac{1}{2}k{\left(\frac{i}{h}\right)}^{2}=\frac{1}{2}\frac{k}{h^{2}}i^{2}$$

Using Equation (13), the stiffness *K*_1_ of the lower side mechanism can be obtained as

(14) $$K_{1}=\frac{k}{h^{2}}$$

Since the mechanism is symmetrical at the top and bottom, similarly, the stiffness *K*_2_ of the upper side mechanism can be obtained as

(15) $$K_{2}=K_{1}=\frac{k}{h^{2}}=K$$

From the stiffness expression of the mechanism, it can be seen that the stiffness of the arm of the surgical forceps depends on the hinge stiffness *k* and the forceps mechanism parameter *h*. Since *h* is the dimension of the forceps design requirement configuration, optimization of the hinge rotational stiffness *k* should be considered in stiffness optimization to optimize the stiffness of the surgical forceps.

According to the expression for the hinge rotational stiffness *k*, the influencing factors are the modulus of elasticity, the depth of the hinge, the minimum thickness of the flexible hinge, and the length of the intermediate beam rod of the straight beam-type flexible hinge. In stiffness optimization, only the other three variables are optimally designed because the material modulus of elasticity is a discrete value.

### 4.4. Kinetic Modeling of the Forceps

In order to model the dynamics, the Lagrangian method was used to find the intrinsic frequency of the arm of the surgical forceps. Let *i* be a generalized coordinate vector corresponding to the input force *F*. Thus, the kinetic and potential energies of the mechanism are expressed in terms of the chosen coordinates and their derivatives.

Firstly, the kinetic energy of the lower lateral forceps arm is shown in Equation (16).

(16) $$\left\{ \begin{array}{l} T_{1}=\frac{1}{2}J_{1}{\dot{\theta }}^{2}\\ J_{1}=m_{1}r_{1}^{2} \end{array}\right.$$

where *J*_1_ is the rotational inertia of the lower arm of the forceps around the straight beam-type flexible hinge; *m*_1_ is the mass of the lower side forceps arm; and *r*_1_ is the distance between the center of mass of the forceps arm and the rotation axis.

Meanwhile, based on Equation (16), the kinetic energy of the lower side forceps arm can be written as

(17) $$T_{1}=\frac{1}{2}m_{1}^{\prime }{\dot{i}}^{2}$$

where $m_{1}^{\prime }=m_{1}\frac{r^{2}}{h^{2}}$.

Furthermore, assuming that the potential energy comes entirely from elastic deformation, the elastic potential energy of the lower lateral forceps arm can be introduced as

(18) $$V_{1}=\frac{1}{2}K_{1}i^{2}$$

Substituting the kinetic energy *T*_1_ and potential energy *V*_1_ into the following Lagrange equation yields Equation (19).

(19) $$\frac{d}{dt}\left(\frac{\partial T_{1}}{\partial \dot{i}}\right)-\frac{\partial T_{1}}{\partial i}+\frac{\partial V_{1}}{\partial i}=F$$

Thus, a general expression for the dynamics can be obtained as

(20) $$m_{1}^{\prime }i+K_{1}i=F$$

The generalized solution of the above equation is the intrinsic frequency of the lower-side forceps arm in the output direction, as shown in Equation (21).

(21) $$f_{1}=\frac{1}{2\pi }\sqrt{\frac{K_{1}}{m_{1}^{\prime }}}$$

Substituting Equation (14) into Equation (21) gives the intrinsic frequency of the lower side forceps arm in the output direction as

(22) $$f_{1}=\frac{1}{2\pi r}\sqrt{\frac{k_{1}}{m_{1}}}$$

After analysis, it can be seen that the other parts of the lower side mechanism only affect the mass distribution of the lower side mechanism without affecting the stiffness, so from Equation (22), the intrinsic frequency of the lower side mechanism in the output direction *f*_1_′ is

(23) $$f_{1}^{\prime }=\frac{1}{2\pi r}\sqrt{\frac{k_{1}}{M}}$$

where *M* is the total mass of the lower side mechanism.

Since the mechanism is symmetrical at the top and bottom, using the same reason, the intrinsic frequency *f*_2_′ of the mechanism at the top side can be obtained as

(24) $$f_{2}^{\prime }=f_{1}^{\prime }=\frac{1}{2\pi r}\sqrt{\frac{k_{1}}{M}}$$

### 4.5. Modal Optimization of the Forceps

Based on the analysis, it can be seen that the fundamental frequency of the up/down mechanism is the fundamental frequency of the surgical forceps. The material used in the mechanism is high-toughness resin with a modulus of elasticity *E* = 2500 MPa, Poisson’s ratio *μ* = 0.3, density *ρ* = 1.117 g/cm^3^, and the volume can be measured by UG.

Based on the expression of the intrinsic frequency of the mechanism, it can be seen that the fundamental frequency of the forceps arm depends on the hinge stiffness and the forceps mass. Based on the structural design, it can be seen that the hinge depth is a factor that determines the forceps mass, and because of a certain forceps material, there exists a certain functional relationship between the forceps mass and the hinge depth.

According to the expression for the hinge rotational stiffness *k*, the influencing factors are the modulus of elasticity, the depth of the hinge, the minimum thickness of the flexible hinge, and the length of the intermediate beam rod of the straight beam-type flexible hinge. Therefore, in the forceps arm fundamental frequency optimization, only the other three variables need to be optimally designed since the material properties are discrete values.

By designing a reasonable finite number of trials by means of response surface analysis, a mathematical model including the primary term of each significant factor, the squared term, and the first-level interaction term between any two factors is developed to study the precise relationship between the factors and the response values and to determine quickly the optimal conditions for the multifactorial system.

Firstly, in Design-Expert, the Box–Behnken method was used to design the hinge depth, hinge minimum thickness, and hinge intermediate beam rod length as the influencing factors, and then the fundamental frequency was designed as the response factor. The design data given by Design-Expert were combined with PATRAN 2010 and NASTRAN 2010 for modal analysis. The design data, modal theory calculation results, and PATRAN 2010 and NASTRAN 2010 modal analysis results are shown in Table 2.

In order to verify the reasonableness of the theoretical calculations, the above table summarizes the results of the comparison between the theoretical values of the first-order modes of the mechanism and the finite element simulation values. It can be seen that the maximum error is 13.81%, and the two main reasons for the large error are as follows: the flexible mechanical structure itself and the displacement loss caused by the elongation of the flexible hinge.

**Figure 7:**
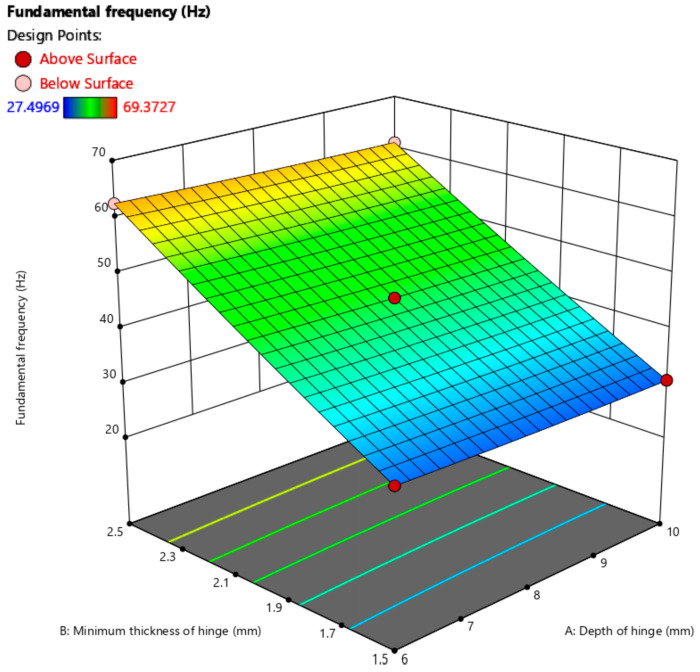
Three-dimensional response surface of the fundamental frequency.

Based on the forceps arm configuration parameters, it can be seen that the length of the middle beam rod of the hinge is 8 mm. The 3D response surface of the fundamental frequency of the surgical forceps was finally obtained, as shown in Figure 7. In Figure 7, it can be seen that the effect of the minimum thickness of the hinge on the fundamental frequency is much larger than the effect of the hinge depth on the forceps’ fundamental frequency.

**Figure 8:**
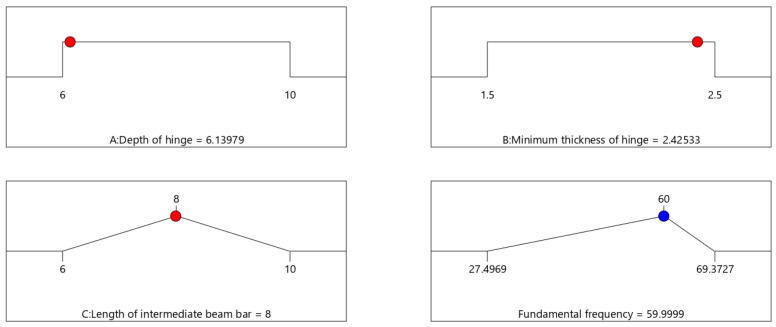
Optimal values of hinge parameters.

During surgical procedures, various involuntary hand movements, including physiological tremors and twitching, can hinder the surgeon’s operation and seriously affect surgical precision. The frequency of physiological tremors of the human hand is 8–12 Hz, and considering the influence of the motor and ball screw, the fundamental frequency of this surgical forceps arm is designed to be 60 Hz. In Design-Expert, the optimal values of hinge depth and minimum thickness of the hinge can be obtained by designing the response target fundamental frequency as 60 Hz, as shown in Figure 8.

**Figure 9:**
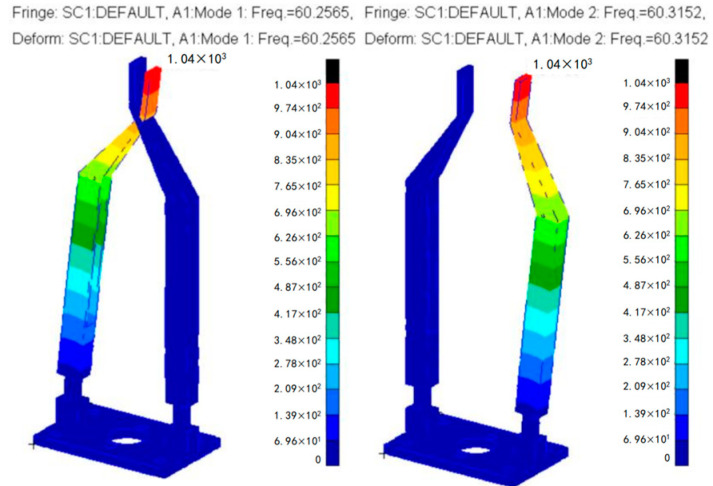
Fundamental frequency in two limit states of the forceps.

The modal analysis of the surgical forceps arm designed using optimal values was carried out in PATRAN 2010 and NASTRAN 2010. The fundamental frequencies are 60.2565 Hz and 60.3152 Hz, as shown in Figure 9. Its maximum error concerning the target fundamental frequency is only 0. 5253%.

**Figure 10:**
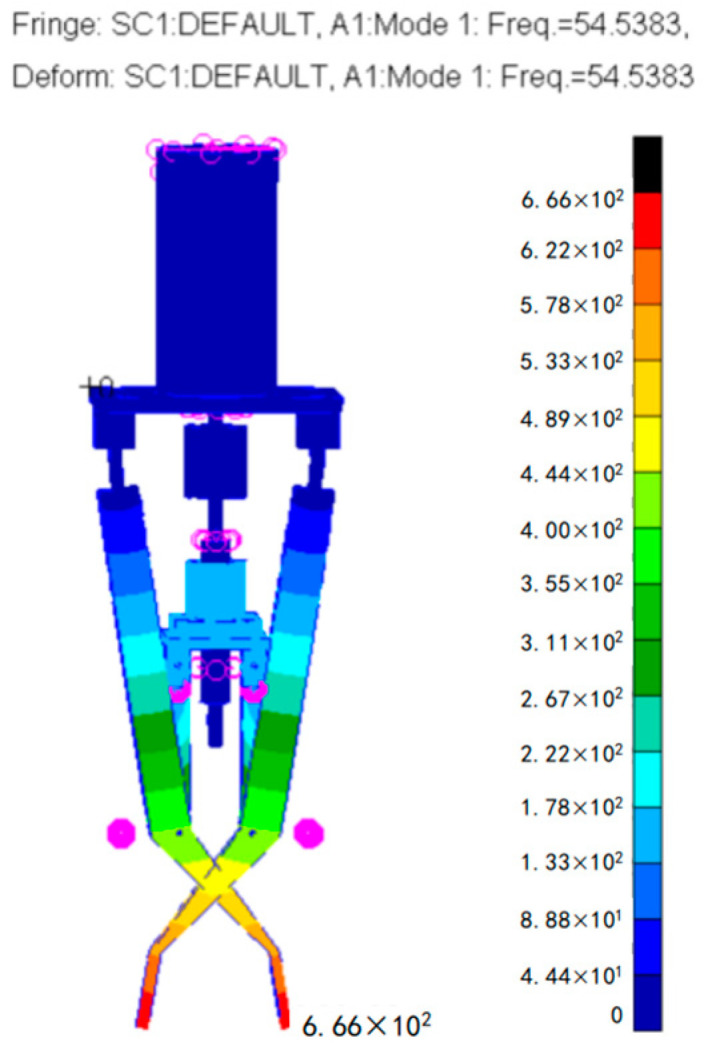
Fundamental frequency of the forceps.

Finally, the modal analysis of the optimized surgical forceps was carried out in PATRAN 2010 and NASTRAN 2010. As shown in Figure 10, it can be concluded that its fundamental frequency is 54.5383 Hz. Since the physiological hand tremor frequency is 8–12 Hz, the fundamental frequency of the optimized surgical forceps reaches 4.5 times the physiological hand tremor frequency, and this fundamental frequency allows the mechanism to respond quickly. This indicates that the designed surgical forceps have good performance and can achieve a high-precision clamping action.

## 5. Experiments

**Figure 11:**
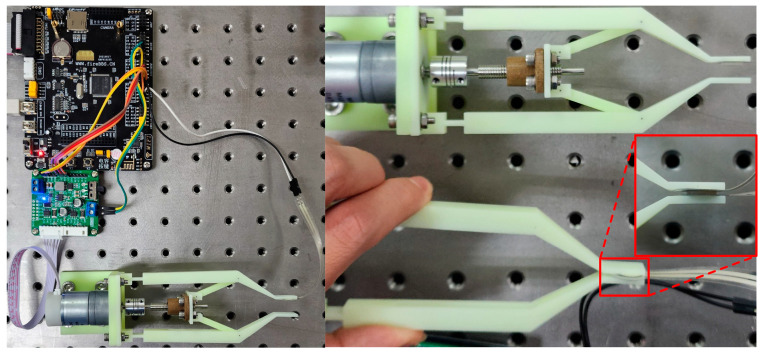
Experimental test platform.

Biomechanical compatibility and safety issues exist between surgical forceps and human tissues because of the lack of a force retention link. Biomechanical compatibility refers to the nature and ability of surgical instruments to match the elastic deformation of tissues when producing mechanical effects between the instruments and biological tissues, i.e., the ability to improve the safety of surgical operations while ensuring the effectiveness of surgical operations. Too much clamping force of the instrument on a tissue during surgical execution is likely to cause unnecessary losses and lead to complications and other adverse reactions, while too little clamping force does not allow for effective execution of surgical operations. Most current hand-held robots solve this problem through single-force feedback, but due during lengthy surgical procedures, this can lead to hand fatigue in the surgeon due to prolonged clamping, which can lead to new problems. In order to solve the problem of the clamping accuracy of surgical forceps, this paper investigates a hand-held surgical robotic forceps with force control and force retention functions. To verify the performance of the new surgical forceps, a principle prototype of the surgical forceps is processed and fabricated in this paper, and the experimental device is designed and fabricated, as shown in Figure 11. Because of the conditions, professional sterilization of these surgical forceps was limited. In the future, when this surgical forceps is applied to surgical procedures, the sterilization of the surgical forceps will be a critical part of ensuring the safety of the procedure, and strict processes and standards will be followed.

The forceps and experimental tooling are made of high-tenacity resin. The forceps clamping force **F** is displayed in the form of mass, which is measured by a resistive thin-film pressure transducer with a range of 50–2000 g pasted on the inner side. Because of the limitations of the conditions, the ability to provide real samples was restricted, so in the experiments, a silicone material, which is closer to human tissues, was chosen instead of human tissues. A silicone ring section with a diameter of 10 mm and a thickness of 1 mm was chosen as the clamping test material.

In order to illustrate the contribution of the designed surgical forceps to the biomechanical compatibility problem, pressure acquisition was carried out with the force-holding mode of the surgical forceps on and off. The experiments were first performed with the force grip mode on by setting the target gripping force of the surgical forceps to 55 g, 65 g, 75 g, and 85 g. The contact force between the jaws and the silicone rubber tissue was recorded in real time from when the target gripping force was reached. Subsequently, the clamp force mode was switched off, and the clamp force experiments were performed with visual feedback from the computer readings at 55 g, 65 g, 75 g, and 85 g of clamp force.

**Figure 12:**
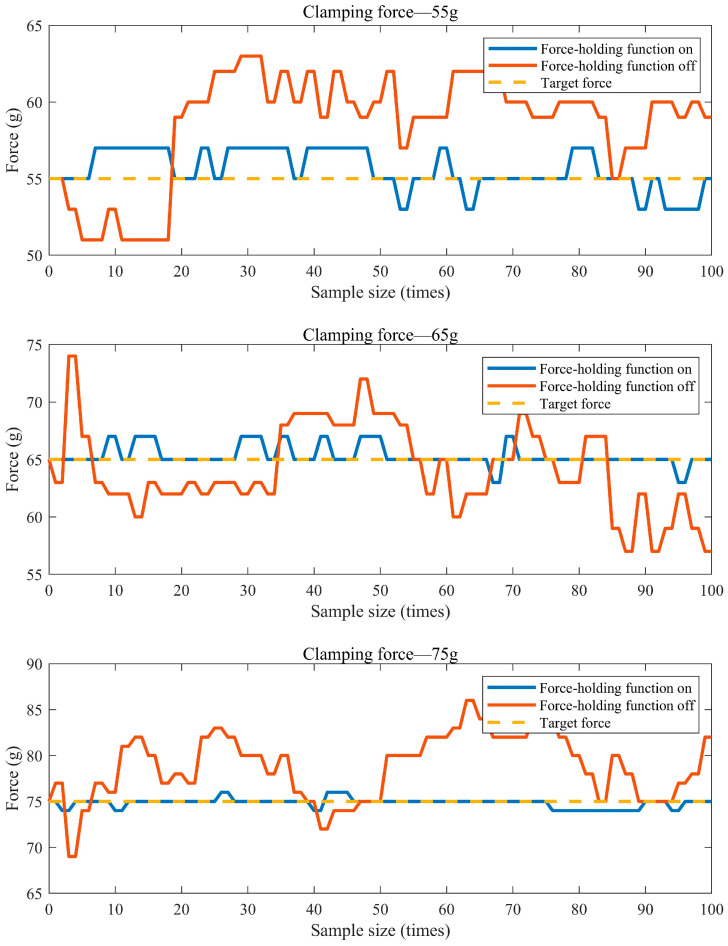
Variation in the gripping force of the forceps.

**Figure 13:**
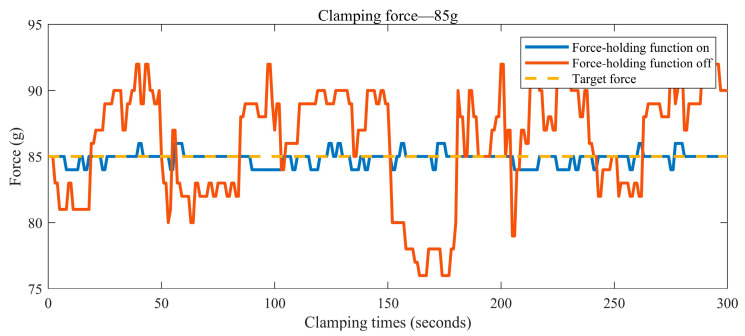
Variation of clamping force with time for long clamping times.

The graph in Figure 12 shows the curve of the contact force between the clamp tip and the silicone tissue as a function of the number of recordings when the surgical clamp force-hold function was switched on and off during the experiment. As can be seen in Figure 12, when the pressures required in the experiment were 55 g, 65 g, and 75 g, the contact force between the surgical forceps and the tissue fluctuated considerably when the force-hold mode was switched off and could not be maintained near the target gripping force. In contrast, when the force-holding mode of the surgical forceps was on, the contact force of the jaws was more centrally maintained near the target clamping force. In particular, the advantages of the designed surgical forceps became more apparent when the target clamping force was greater. The graph in Figure 13 shows the curve of the contact force between the jaws and the silicone tissue over time when the surgical force-holding function of the surgical forceps was switched on and off, respectively, during the experiment. As can be seen in Figure 13, when the target clamping force was 85 g, the maximum error between the actual clamping force and the target clamping force reached 9 g within five minutes when the force-hold function was switched off. When the force-hold function was switched on, the maximum error between the actual clamping force and the target clamping force was 1 g, and the maximum error was only 11% of that when the force-hold function was switched off. Therefore, the clamping accuracy of the surgical forceps was greatly improved when the force-hold function was switched on. It can also be seen in Figure 13 that the force-hold function of these surgical forceps has good stability over time. The experimental results show that compared with the surgical forceps in other articles, the force-holding function of these surgical forceps can solve the problem of surgeon hand fatigue due to prolonged clamping and improve the clamping precision of surgical forceps. Meanwhile, the designed surgical forceps have good stability and are suitable for long-term surgical procedures. Therefore, the new surgical forceps meet the design expectations.

## 6. Conclusions

In this paper, a new type of surgical forceps with force control and force retention functions was investigated to address the problem that physiological hand tremors and twitching as well as the nonlinear relationship between the surgical forceps gripping force and the operating force seriously affect the gripping accuracy of surgical instruments. Unlike other surgical forceps, the surgical forceps designed in this study have a force-holding function, which can reduce a surgeon’s hand fatigue during surgery. At the same time, optimizing the fundamental frequency of the surgical forceps from the root can prevent the resonance phenomenon of surgical forceps with physiological hand tremors to improve the clamping accuracy. The surgical forceps adopt a flexible hinge design with a simple and compact structure and achieve displacement amplification and motion transfer through the lever principle. The simulation test results show that the simulated values are very close to the theoretical model within the specified working range, and its maximum error is 2.56%. By establishing the kinetic model of the surgical forceps and optimizing its design, the fundamental frequency of surgical forceps is found to be 54.5383 Hz, which reaches 4.5 times the physiological tremor frequency of the human hand. Finally, the optimized surgical forceps were experimentally verified, and the results show that the surgical forceps can control the clamping force within the safety threshold, which greatly improves the stability of the surgical clamping force, thus playing a protective role. The designed surgical forceps have good clamping performance and can achieve high-precision clamping and unclamping operations, which verifies the effectiveness of the surgical forceps. The results of the related study can ensure the stability of the clamping force when surgeons use surgical forceps to clamp tissues, thus improving the clamping accuracy of the surgical forceps. This is important for improving the biomechanical compatibility of surgical instruments and the safety of surgical clinical practice.

## Figures and Tables

**Table 1:** Conformational parameters of the surgical forceps.

Name of the Configuration	Dimensions
*a*(BD)	30.6425 mm
*b*(OD)	72.0156 mm
*c*(OI)	12.5000 mm
*d*_0_(BH)	43.4000 mm
*f*(DF)	43.8141 mm
*g*(OF)	114.2520 mm
*h*(IF)	113.0000 mm
*j*(OA)	40.0000 mm
*α*_0_(∠BOD)	14.9267°
*β*_0_(∠AOB)	73.8798°
*γ*_0_(∠AOF)	81.4430°
*e*(OB)	Awaiting an answer
*j*(OA)	Awaiting an answer
*s*(FG)	Awaiting an answer

**Table 2:** Theoretical and simulation fundamental frequencies of various hinges.

Group	*n*/mm	*p*/mm	*l*/mm	Theory/Hz	Simulation/Hz
s1	6	2.5	8	64.0059	62.5141
2	6	2	10	39.1285	41.6050
3	6	2	6	55.3639	52.9845
4	6	1.5	8	29.8883	31.5355
5	8	2.5	10	56.4905	55.5250
6	8	2	8	46.9276	45.7590
7	8	1.5	10	26.4002	27.4969
8	8	1.5	6	36.8082	35.8030
9	8	2.5	6	78.9533	69.3727
10	10	2	6	57.3819	51.8664
11	10	2.5	8	66.3492	61.0414
12	10	2	10	41.0959	40.5387
13	10	1.5	8	30.9777	30.6796

## Data Availability

The data presented in this study are available on request from the corresponding author.
